# Supplementary material for: Calcium-sensing receptor-mediated NLRP3 inflammasome response to calciprotein particles drives inflammation in rheumatoid arthritis
Source: Nat Commun. 2020 Aug 25;11:4243. doi: 10.1038/s41467-020-17749-6 (PMC7447633; doi:10.1038/s41467-020-17749-6)
Supplement: Supplementary file 1 — Supplementary Information [file 41467_2020_17749_MOESM1_ESM.pdf]

Supplementary Information

# Calcium-sensing receptor-mediated NLRP3 inflammasome response to calciprotein particles drives inflammation in rheumatoid arthritis

Jäger, Murthy et al.

Corresponding authors:

Manuela Rossol: manuela.rossol@medizin.uni-leipzig.de

Ulf Wagner: ulf.wagner@medizin.uni-leipzig.de

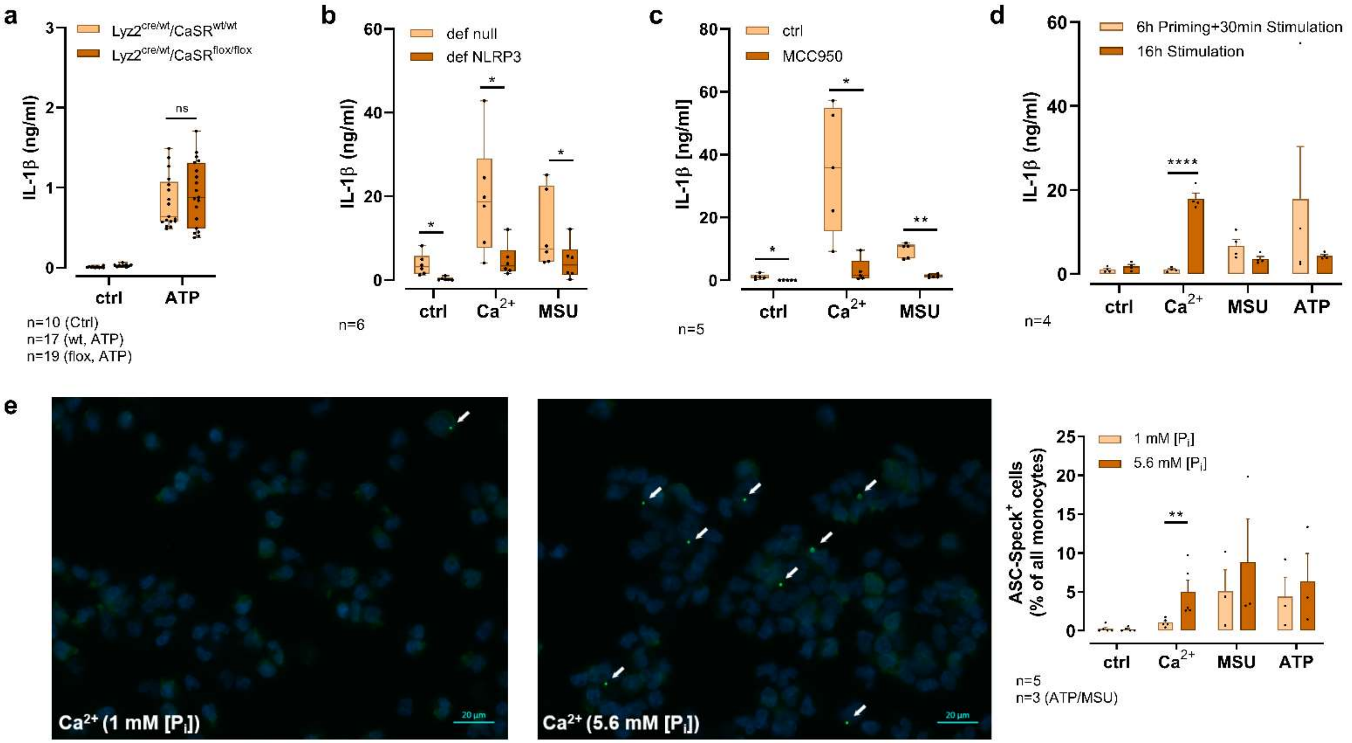


**Supplementary Figure 1:**

**a** – Treatment of mouse blood monocytes of myeloid CaSR-KO mice (B6.129P2-Lyz2$^{tm1(cre)Ifo7j}$xCaSR$^{\Delta flox/\Delta flox}$) or control mice (B6.129P2-Lyz2$^{tm1(cre)Ifo7j}$xCaSR$^{WT/WT}$, n=17, 19) with 3mM ATP and concomitant LPS treatment for 16h compared to controls (ctrl). Differences not statistically significant. **b** – Differentiated NLRP3-deficient (def NLRP3) or control (def null) THP-1 cells were LPS-primed and incubated with 2.5mM added [$Ca^{2+}$], or 100µg/ml MSU in RPMI1640/10%FBS containing 5.6mM [Pi] for 16h (n=6). Statistical analysis was performed using two-tailed paired t test. **c** – Human primed monocytes were treated with LPS and either 2.5mM added [$Ca^{2+}$] or 100µg/ml MSU for 16h in the presence of the NLRP3 inhibitor MCC950 (n=5). Statistical analysis was performed using two-tailed paired t test. **a-c** - IL-1β was detected in supernatants after 16h. Data are shown as box-and-whisker plot with median, 25-75$^{th}$ percentile, and min/max whiskers. P-values are indicated as * $p<0.05$, ** $p<0.01$, *** $p<0.001$, **** $p<0.0001$. **d** – Treatment of monocytes with 2.5 mM added [$Ca^{2+}$]; 100 µg/ml MSU crystals, or 3mM ATP compared to controls (ctrl) either after 6 hours of priming with LPS and subsequent 30min of stimulation or after stimulation and concomitant LPS treatment for 16h (n=4). Data are shown as mean values ± s.e.m. Statistical analysis was performed using unpaired t test. **e** – Human primed monocytes were treated with LPS and either 2.5mM added [$Ca^{2+}$] (n=5), 3mM ATP (n=3), or 100µg/ml MSU (n=3) at the indicated [$P_i$] for 8h. Monocytes were fixated, and ASC was detected by immunofluorescence staining (green). DNA was stained with Hoechst (blue). Representative immunofluorescence images show [$Ca^{2+}$]-induced ASC speck formation at 1mM and 5.6mM [$P_i$]. Bar charts show percentage of ASC-speck-positive cells for each condition. Data are shown as mean values ± SEM. Statistical analysis was performed using two-tailed Mann-Whitney U test.

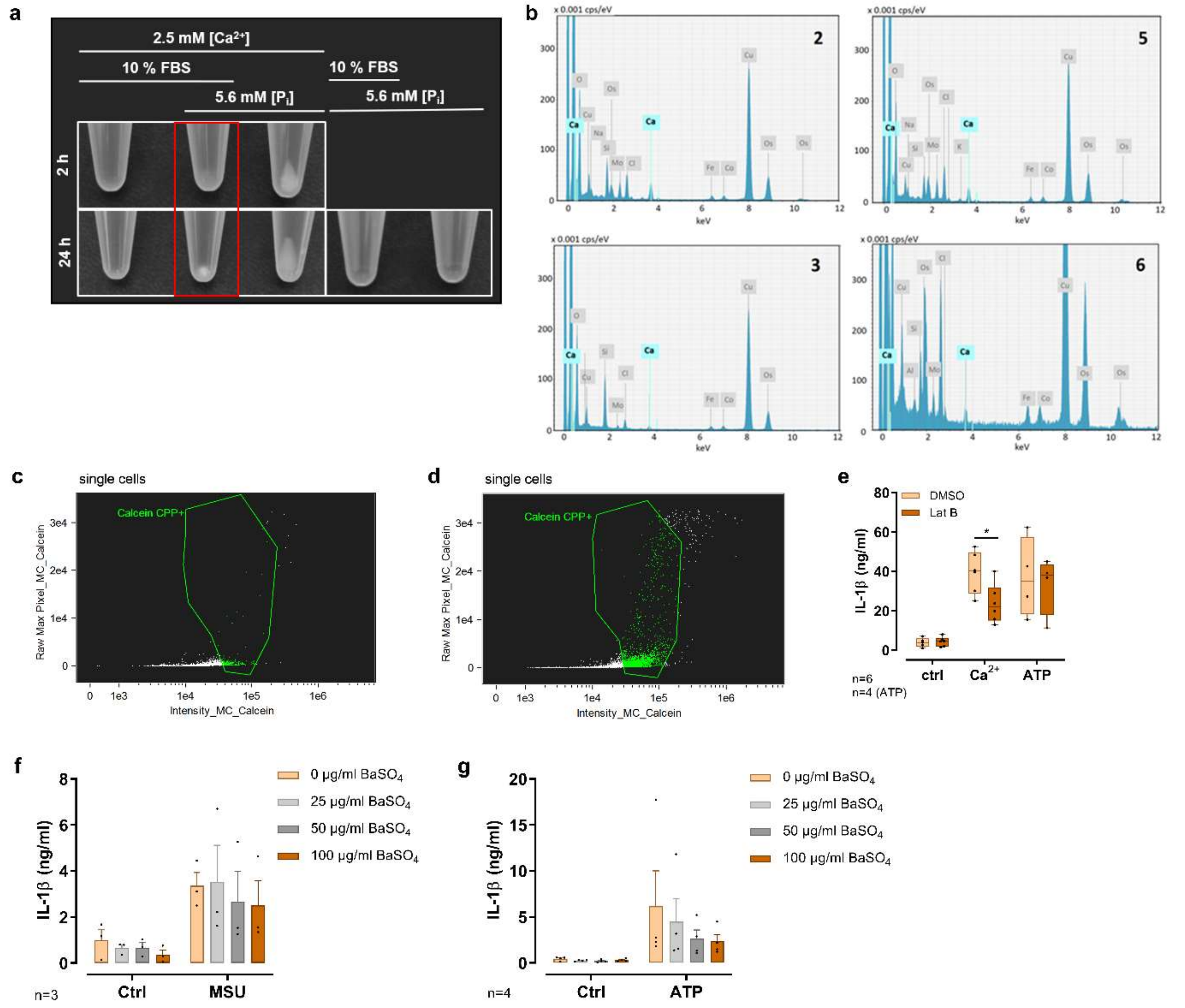


**Supplementary Figure 2:**

**a** – Sedimentation of spontaneously formed particles in RPMI1640 in the presence or absence of 10% FBS, 2.5 mM added [$Ca^{2+}$], and/ or 5.6 mM [$P_i$], as indicated. Pellets were sedimented by centrifugation for 2 h at 16,000 x g at 21°C. If no [$P_i$] is stated, 1 mM [$P_i$] is present in the medium; **b** – EDX spectra for labeled regions in figure 1l; **c,d** – Gating strategy for imaging flow cytometry measurements and analysis of calcein-uptake of LPS-primed (b) and $Ca^{2+}$-stimulated monocytes (c); **e** – Detection of IL-1β via ELISA in cell culture supernatants after 16h of incubation following pre-incubation with 500nM Latrunculin B (Lat B) or the according DMSO dilution (DMSO) prior to stimulation with 2.5mM added [$Ca^{2+}$] (n=6) or 3mM ATP (n=3) in RPMI1640/10%FBS containing 5.6mM [$P_i$]. Statistical analysis was performed using two-tailed Mann-Whitney U test. Data are shown as box-and-whisker plot with median, 25-75$^{th}$ percentile, and min/max whiskers. P-value is indicated as * $p<0.05$. **f,g** – Treatment of monocytes with the indicated concentrations of albumin-coated $BaSO_4$ nanoparticles in the absence (ctrl) or presence of 100µg/ml MSU crystals for 16 h (f, n=3) or after 6h of LPS priming followed by 30min stimulation with $BaSO_4$ nanoparticles with or without 3mM ATP (g, n=4). Data are shown as mean values ± SEM. Differences not statistically significant.

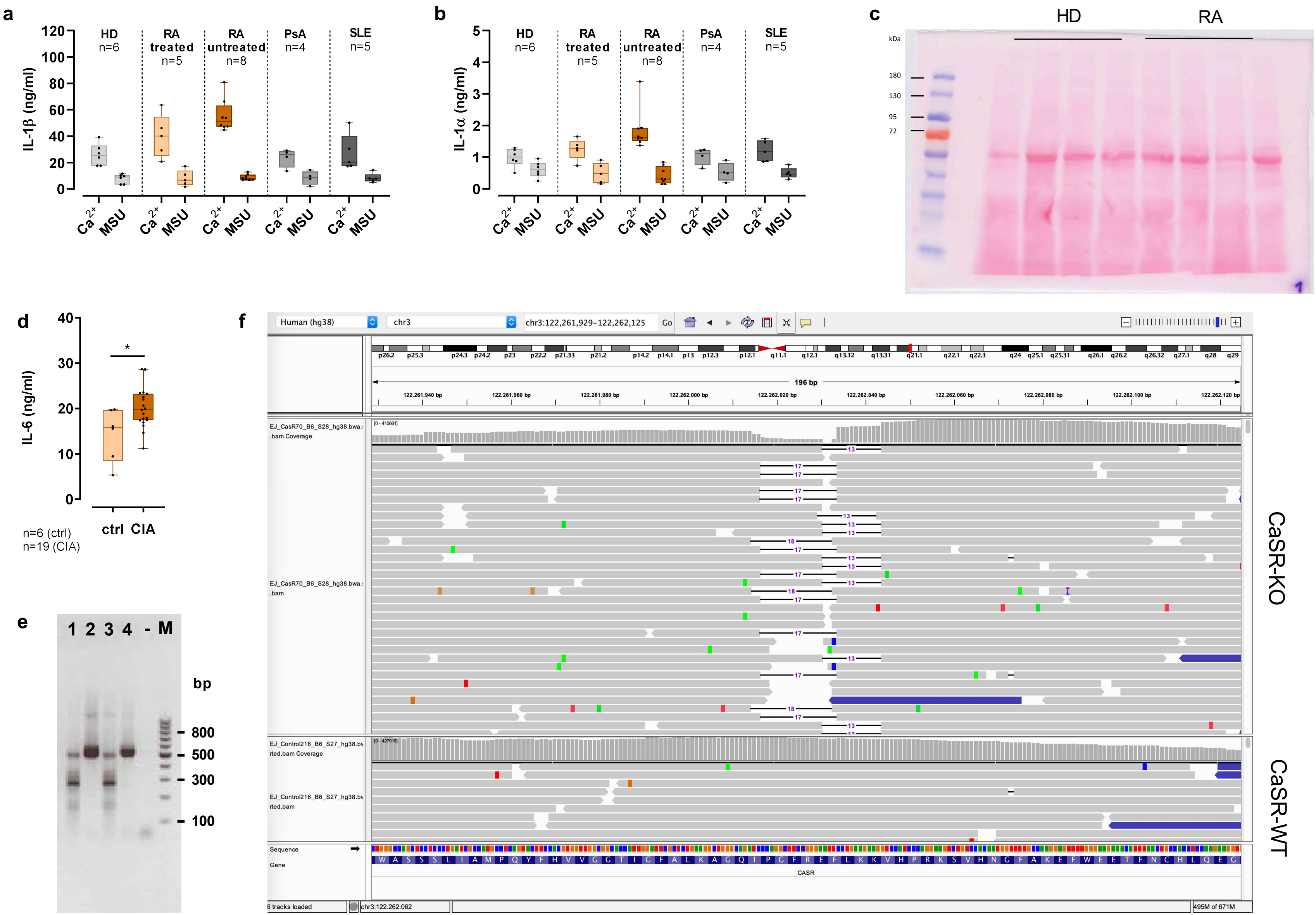
a
IL-1β (ng/ml)
HD
n=6
RA
treated
n=5
RA
untreated
n=8
PsA
n=4
SLE
n=5
Ca2+
MSU
b
IL-1α (ng/ml)
c
HD
RA
kDa
180
130
95
72
d
IL-6 (ng/ml)
*
ctrl
CIA
n=6 (ctrl)
n=19 (CIA)
e
1 2 3 4 - M
bp
800
500
300
100
f
Human (hg38)
chr3
chr3:122,261,929-122,262,125
Go
196 bp
EJ_CasR70_B6_S28_hg38.bwa.
.bam Coverage
EJ_CasR70_B6_S28_hg38.bwa.
.bam
EJ_Control216_B6_S27_hg38.bv
rted.bam Coverage
EJ_Control216_B6_S27_hg38.bv
rted.bam
Sequence
Gene
CASR
6 tracks loaded
chr3:122,262,062
495M of 671M
CaSR-KO
CaSR-WT

**Supplementary Figure 3:**

**a,b** – Detection of IL-1β (a) and IL-1α (b) in cell culture supernatants of LPS-primed, freshly isolated peripheral blood monocytes from patients with treated RA (n=5), untreated RA (n=8), psoriatic arthritis (PsA, n=4), systemic lupus erythematodes (SLE, n=6) or healthy controls (HC, n=6) after 16h of stimulation with 2.5mM added [$Ca^{2+}$] or 100µg/ml MSU crystals in RPMI1640/10%FBS containing 5.6 mM [$P_i$]. Data are shown as box-and-whisker plot with median, 25-75$^{th}$ percentile, and min/max whiskers. Differences not statistically significant. **c** – Ponceau staining of PVDF membrane from the CaSR Western Blot shown in figure 4g, whole lanes from Ponceau staining were used for calculating relative CaSR protein expression in monocytes from healthy donors (HD) or RA patients (RA). Shown is one experiment out of two. **d** – IL-6 secretion after 16h incubation with 2.5mM added [$Ca^{2+}$] of $CD11b^+$ mononuclear cells from bone marrow of mice with CIA (n=19) and control animals (n=6). Data are shown as box-and-whisker plot with median, 25-75$^{th}$ percentile, and min/max whiskers. Statistical analysis was performed using two-tailed Mann-Whitney U test. P-value is indicated as * $p<0.05$. **e,f** – Visualization of CRISPR-Cas9 induced mutational pattern of the CaSR gene in THP-1 cells as reported previously[1]. **e** – Results of mismatch assay, PCR amplicon of the Cas9 target region (exon 4) was digested with resolvase and loaded onto an agarose gel. Shown are results from two different culture passages. Selected clones are transduced with non-sense sgRNA (lanes 2+4) and with CaSR targeted sgRNA (lanes 1+3). $H_2O$ control (-); gene ruler 100-bp DNA ladder (M). **f** – The sequencing reads and coverage for clone CaSR70_B6 compared to wildtype. Sequencing (n=1) reveals a compound heterozygote with three alleles all containing deletions of 13, 17 or 18 bp at the edited sites. Since THP-1 cells are known to contain numerous aberrations of insertions and deletions, including chromosome 3, and in view of local selective variations occurring in most labs using the cell line over prolonged periods of time, the occurrence of three alleles is not unexpected.[2]

Supplementary References

1. Bell, C. C., Magor, G. W., Gillinder, K. R. & Perkins, A. C. A high-throughput screening strategy for detecting CRISPR-Cas9 induced mutations using next-generation sequencing. *BMC Genomics* (2014).
2. Adati, N., Huang, M. C., Suzuki, T., Suzuki, H. & Kojima, T. High-resolution analysis of aberrant regions in autosomal chromosomes in human leukemia THP-1 cell line. *BMC Res. Notes* (2009).
